# Modular stem in total hip arthroplasty for patients with trochanter valgus deformity: surgical technique and case series

**DOI:** 10.1186/s12891-020-3145-4

**Published:** 2020-02-24

**Authors:** Xiangpeng Kong, Wei Chai, Minzhi Yang, Alvin Ong, Jiying Chen, Yan Wang, Yonggang Zhou

**Affiliations:** 1 Chinese PLA General Hospital, No.28 Fuxing Road, Haidian, Beijing, China; 2 Nankai university, Tianjin, China; 3 The Rothman Institute, Thomas Jefferson University, 500 English Creek Avenue, Building 1300, Egg Harbor Township, Philadelphia, USA

**Keywords:** Cementless modular stem, Reverse sleeve, Total hip arthroplasty, Trochanter valgus deformity

## Abstract

**Background:**

Trochanter valgus deformity (TVD) is a rare condition of total hip arthroplasty (THA). Femoral osteotomy could be required in correcting the deformity to implant femoral stem in severe TVD. In this study, we described one unpublished technique of reverse sleeve of S-ROM to get through the complex situation. This study aimed to summarize and evaluate its technical challenges, safety and effectiveness.

**Methods:**

From January 2006 to December 2014, we enrolled patients whose sleeves were implanted towards the great trochanter in THA with TVD. Their demographics, perioperative and postoperative information were recorded. To explore its indication, we measured and analyzed the ratio of greater trochanter/lesser trochanter (G/L ratio) and trochanter valgus angle (TVA).

**Results:**

Twelve patients (1 male and 11 female, average age 42.30 ± 10.23) had mean follow-up of 6 years. Among them, only two patients had intraoperative femoral fracture. The survivorship of femoral prosthesis was 100%. The Harris hip score (HHS) increased from preoperative 34.31 ± 14.43 to postoperative 84.12 ± 11.33. All patients’ G/L ratio were larger than 1.50.

**Conclusions:**

The reverse sleeve of S-ROM was a reliable method for the patients with severe TVD, which brought satisfying clinical outcomes in mid-term follow-up.

## Background

Trochanter valgus deformity (TVD) is an uncommon type of proximal femoral deformity [1, 2]. Before widely use of ceramics and highly cross-linked polyethylene in total hip arthroplasty (THA), trochanter valgus osteotomy (TVO) was a useful treatment for developmental dysplasia of the hip (DDH) and osteonecrosis of the femoral head (ONFH) in young patients [3, 4]. When these patients develop severe hip arthritis, they have no choice but hip replacement. The significant angled femoral cavity would complicate femoral preparation and stem implantation [5, 6]. Therefore, THA with concurrent femoral osteotomy is a demanding procedure technically.

Few studies on patients who had TVD have been published [1, 2, 5]. Lewallen et al. reported 32% patients underwent reoperation at 4.6 years after simultaneous THA and femoral osteotomy [5]. Iwase et al. reported that the failure rate of cementless stem was 22.5% at 4 years after conversion THA and concluded that cemented stems were preferable for patients with previous femoral valgus osteotomy [1]. On the other side, another surgeon in 2017 reported 100% survivorship of femoral stem and suggested that modular femoral components should be used when undertaking hip replacement in patients with previous femoral valgus osteotomy [2].

Appropriate type of femoral component could simplify surgical procedures and improve clinical outcomes. The S-ROM femoral component (DePuy Orthopaedics, Warsaw, Indiana) is a cementless, modular and cylindrical prosthesis, which was specially designed for proximal femoral deformity [7]. Modularity at stem-sleeve junction allows surgeon to decide the anteversion of femoral stem independent of sleeve, which could best fit and fill proximal femur.

In this case-series study, we described one special implanting position of sleeve of S-ROM in hip replacement for patients with TVD. Sleeve towards the great trochanter could take advantage of, rather than correct the deformity, which could improve surgical efficiency and reduce trauma greatly. Although this technique has been discussed in some meetings before, no previous studies ever specially described it. This study aimed to summarize and evaluate its technical challenges, safety and effectiveness.

## Patients and methods

The study was approved by the institutional review board. From January 2006 to December 2014, we reviewed 15 patients with sleeves towards the greater trochanter in our joint registry system. Three patients who haven’t had regular follow-up (> 1 years) or complete clinical information were excluded. The remaining 12 patients had severe hip arthritis and TVD.

The S-ROM consists of the sleeve and stem. The sleeve is porouscoated or hydroxyapatite (HA)-coated, and is designed to convert shear and hoop stresses to compressive forces at the sleeve-bone interface. The titanium alloy stem is polished distally; it also has several options of neck length and offset proximally [7]. The sleeve achieves bone in-growth in the metaphysis and the stem can be freely rotated to accommodate any deformity or asymmetry in proximal femur [8].

The angle between sleeve’s spout and stem is 30 degree, which is designed to adapt the medial cortex of proximal femur, so sleeve’s triangle was placed to the lesser trochanter commonly. In this study, we placed the sleeve towards the opposite direction, which means its triangle points to the greater trochanter.

### Surgical technique

In clinical practice, we usually used film (before 2014) or the Orthoview software (Version 6.6.1, Materialise, Leuven, Belgium) for preoperative template. Common femoral stem could not be inserted without corrective osteotomy simultaneously, so we tried to choose S-ROM and implant sleeve towards the greater trochanter. All surgeries were performed by two senior surgeons through posterolateral approach.

**Fig. 1:**
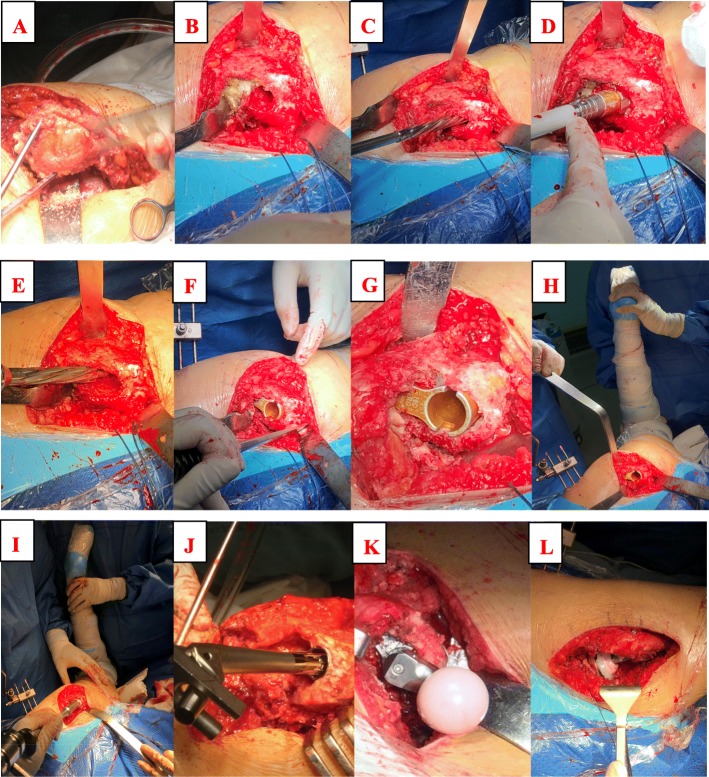
Surgical procedures of implanting the sleeve towards to the greater trochanter. **a**: femoral neck osteotomy. **b**: recognizing the site of femur entry. **c**: reaming the distal femur. **d**: reaming the proximal femur. **e**: preparing the spout of sleeve. **f**: resurfacing the medial cortex. **ghi**: implanting the sleeve towards the greater trochanter. **jkl**: implanting the femoral stem and reduction

1. After dislocating femoral head, femoral neck osteotomy was performed along with the intertrochanteric crest (Fig. 1a).
2. The accurate entry can obtain the proper stem alignment and decrease the risk of periprosthetic fracture. We first located the site of entry according to surgical plan (Fig. 1b). Then the smallest reamer was used to find medullary cavity with or without the aid of intraoperative fluoroscopy (Fig. 1c). Then the distal reamer size was increased sequentially until it touched the cortical bone. The depth of reaming was appropriate when its mark aligned with the peak of the greater trochanter. More attention should be paid to the orientation of reamer to avoid protrusion.
3. In refer to the size of distal reamer, proximal reamer was used to prepare the proximal femoral cavity (Fig. 1d). When the medial cortex of femur is unable to support the sleeve, we placed the sleeve towards the greater trochanter. Because there were no specific tools, the surgeon employed the reamer to prepare the calcar and handled the spout manually (Fig. 1e). The medial cortical bone should be resurfaced to prevent impingement (Fig. 1f). In order to avoid subsidence of sleeve and stem, we adopted larger sleeve as possible (Fig. 1gh).
4. The stem was adjusted in proper anteversion. When the hip stability and leg length were satisfying, the real femoral components were implanted. (Fig. 1ijkl).
5. For the patients who had residual plate and screw, the surgeon should watch out fractures of the greater trochanter. In some cases, wires were pre-bundled around the trochanter and removed after reduction.

### Postoperative follow up and evaluations

The patients were followed up at 4 and 12 months after surgery, and were checked every 2–3 year.

We analyzed the perioperative and postoperative complications, Harris hip score (HHS) and radiographic results in the last follow-up. Complications were defined as neurovascular impairment, dislocation, aseptic loosening, periprosthetic femoral fractures, periprosthetic joint infection and re-operation for any reasons.

**Fig. 2:**
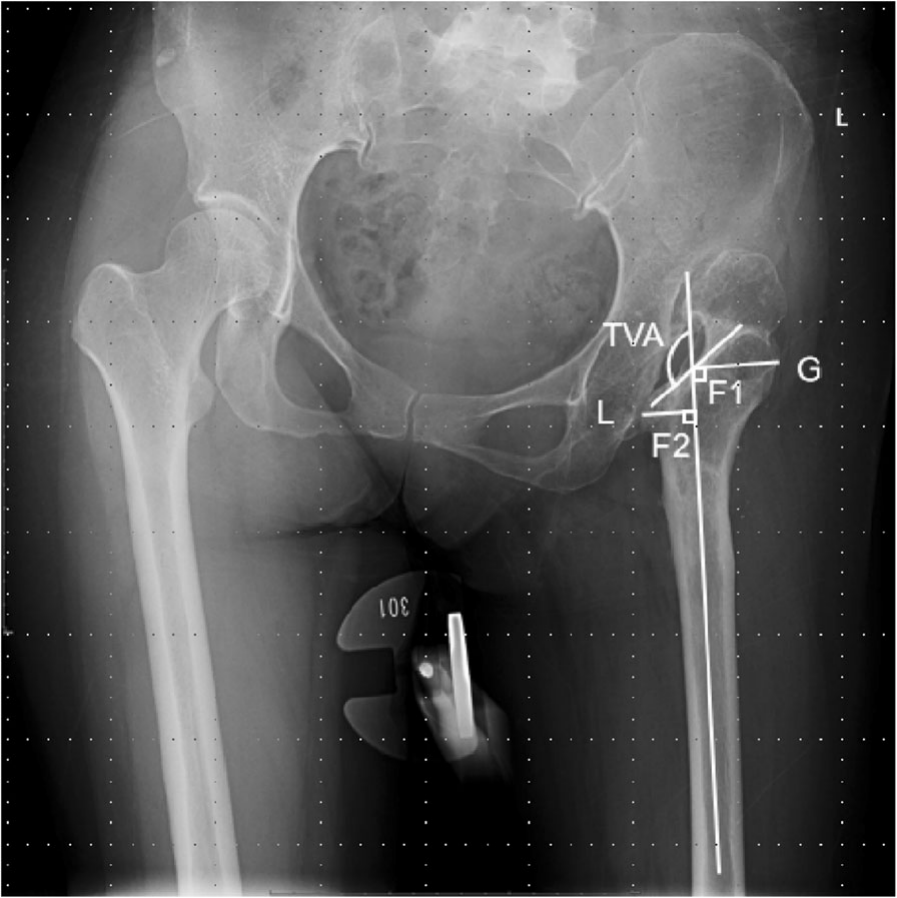
The measurement of G/L ratio and TVA. The midpoints of medullary cavity between the proximal femur (2 cm below the lessor trochanter) and middle femur (10 cm below the lessor trochanter) were connected as the femoral axis. The distance from the peak of greater trochanter to the femoral axis (GF_1_) and the distance from the peak of lessor trochanter to the femoral axis (LF_2_) were measured. G/L ratio = GF_1_ / LF_2_. Trochanter valgus angle (TVA) was defined as the angle between the femoral axis and the intertrochanteric crest

Postoperative radiographic evaluation: migration of the femoral component was assessed by the measurement of the vertical distance from the lower edge of stem to the peak of the greater trochanter, and the angle between the axis of stem and femur. Femoral subsidence of > 4 mm, or swing in the stem alignment of > 2°, or a complete radiolucent line was considered as stem loosening [9–11]. The fixation of proximal sleeve was classified into bone ingrown, fibrous stable, or unstable, according to the classification system of Engh [12]. Spot welding was defined as bone densification and trabecular streaming between the cortex and the implant [10]. The angle of varus stem was marked as positive and valgus stem as negative. The method of measuring the G/L ratio and trochanter valgus angle (TVA) was showed (Fig. 2).

### Statistical analysis

Statistical analysis was performed by SPSS 21.0 statistical software (Inc, Chicago, US). All data were quantitative expressed as *x ± s* (maximum and minimum, median, interquartile range). The test level value *p* is taken as 0.05 on both sides. The intraclass correlation coefficient (ICC) was used to determine variations in different measurements. The radiologic measurement was performed by two independent observers (KXP and YMZ). Each observer made the measurements again after 2 weeks without knowing the first values. The intra-observer and inter-observer agreements were found to have nearly perfect reliability for all of the measurements (ICC > 0.81).

## Results

Twelve patients (11 female and 1 male) were enrolled in this study. Their basic information was showed (Table 1). The primary diagnoses of these patients were DDH (9, 75%), ankylosing spondylitis (1, 8.3%), ONFH (1, 8.3%) and sequela of proximal femoral fracture (1, 8.3%). The causes of TVO were osteotomy (10, 83.33%), suppurative joint sequela (1, 8.3%), malunion of fracture (1, 8.3%).

**Table 1:** The basic information of patients

Patients	Data
Gender (male:female)	1:11
Age (years)	42.30 ± 10.23 (30–66, 45, 15)
BMI (kg/m2)	22.99 ± 2.34 (19.10–30.10, 24.10, 7.88)
Follow up (years)	5.96 ± 3.29 (2–13, 7, 7)
Preoperative Harris score	34.31 ± 14.43 (17–62, 32, 19)
Postoperative Harris score	84.12 ± 11.33 (67–98, 88, 21)

All data were quantitative expressed as x ± s (maximum and minimum, median, interquartile range)

All patients got the neutral alignment of femoral stem (< 3°). There was no neurovascular impairment, dislocation, aseptic loosening, periprosthetic femoral fracture, periprosthetic joint infection or reoperation until the last follow-up.

One patient had a small split in the lesser trochanter and was treated with a cerclage wire. The other patient had fracture in the bottom of the greater trochanter when we removed the previous plate. Frozen cortical strut allograft and titanium cable were used to immobilize the trochanter. The fractures in the two patients were healed within postoperative 4 months.

All 12 hips demonstrated stable bone ingrowth. Spot welding around the inferior border of metaphyseal sleeve was observed in 10 hips (83.33%).

The mean G/L ratios in 12 patient were 2.58 ± 0.95 and all of these were larger than 1.50 (Table 2).

**Table 2:** The comparison of G/L ratio and TVA in two groups

Patients	Data
G/L ratio	2.58 ± 0.95 (1.50–4.43, 2.60, 2.11)
TVA (°)	144.50 ± 9.66 (128–156, 142, 20)

All data were quantitative expressed as x ± s (maximum and minimum, median, interquartile range)

**Fig. 3:**
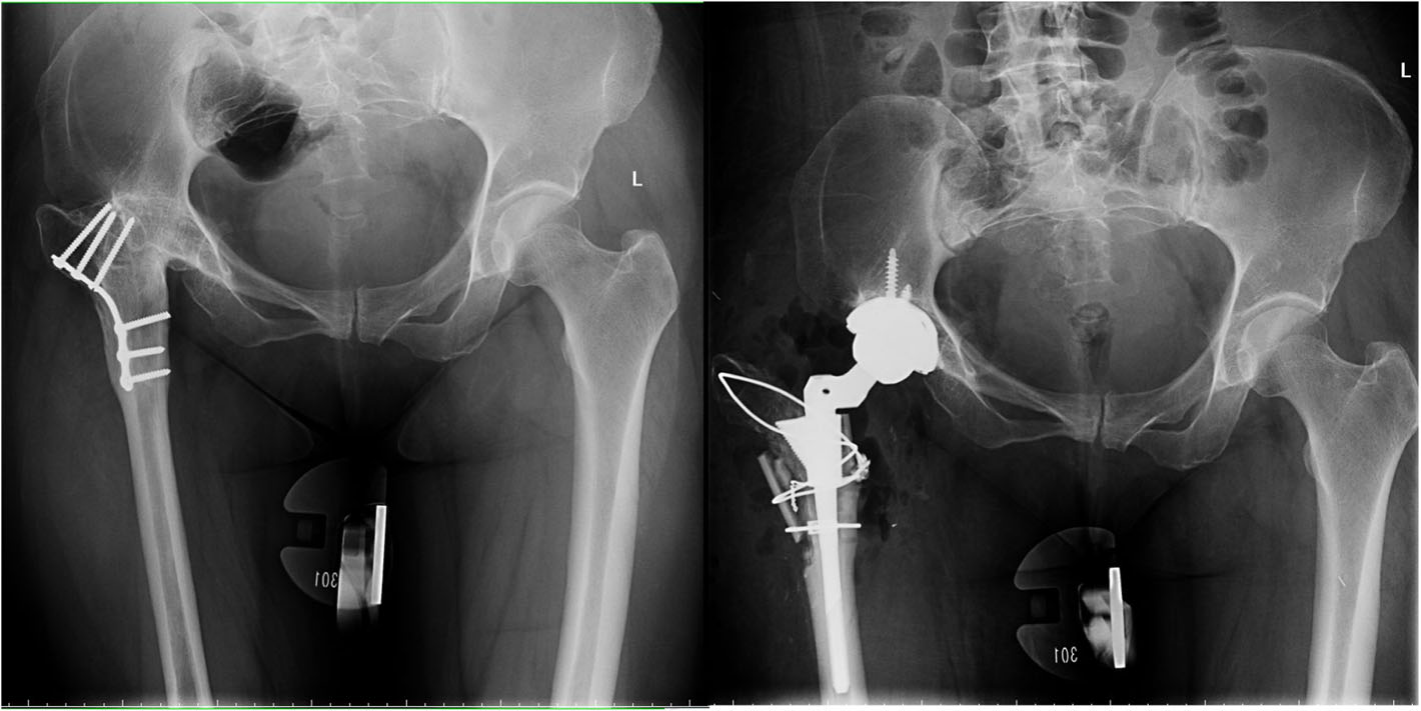
Female, 43 years. She underwent transtrochanteric valgus osteotomy 25 years ago. Her greater trochanter fractured during operation

**Fig. 4:**
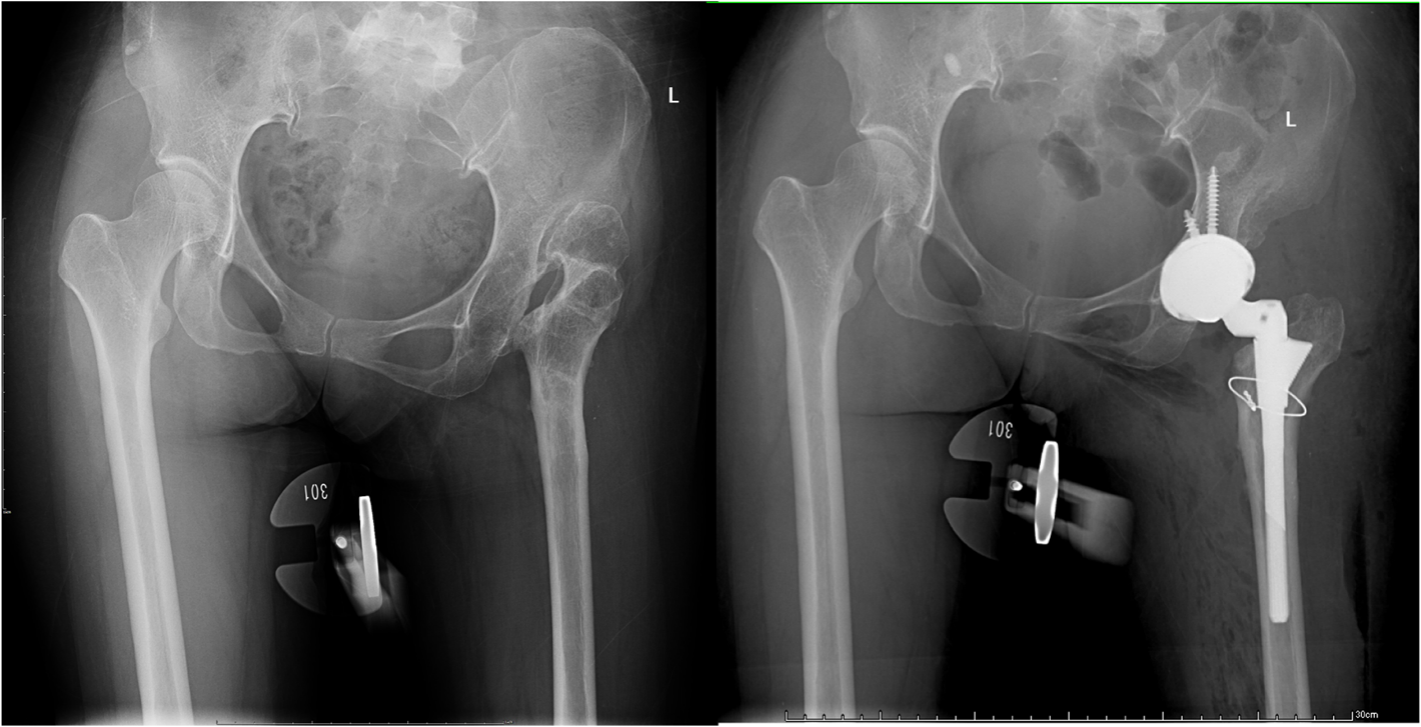
Female, 38 years. She underwent transtrochanteric valgus osteotomy 20 years ago. Her greater trochanter fractured during operation

**Fig. 5:**
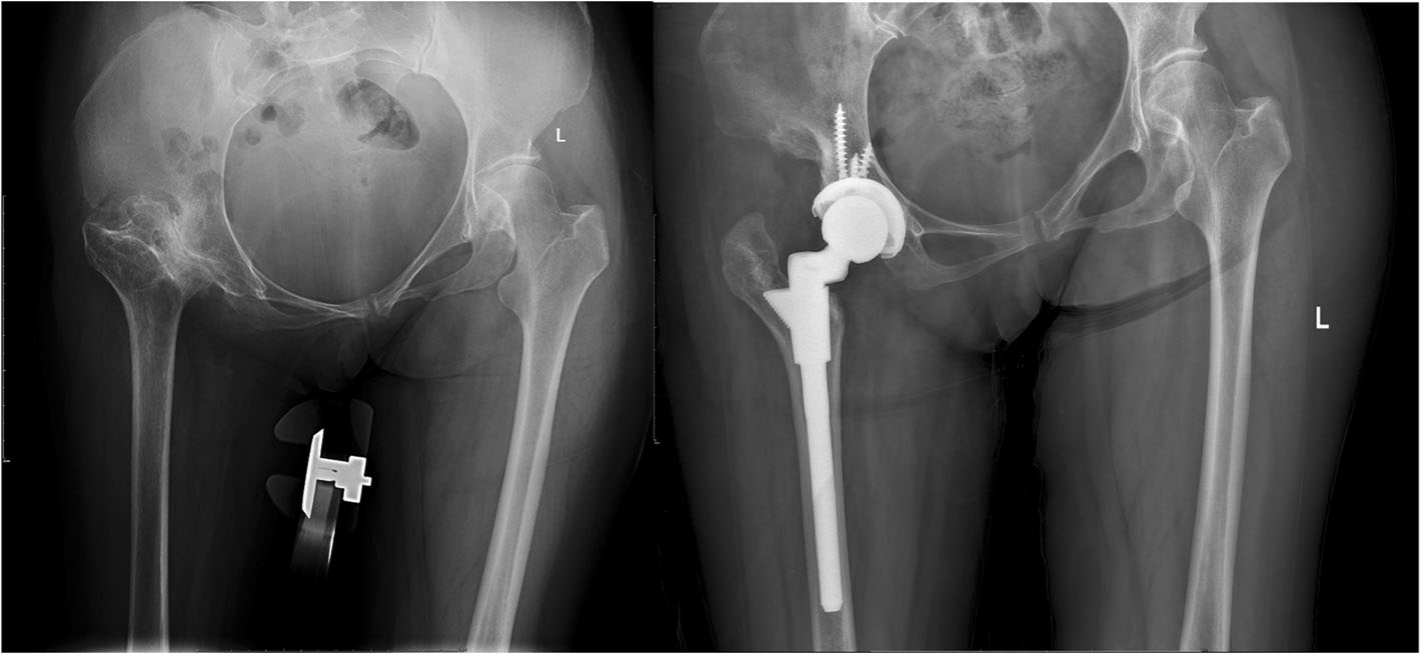
Female, 54 years. She underwent transtrochanteric valgus osteotomy 27 years ago

**Fig. 6:**
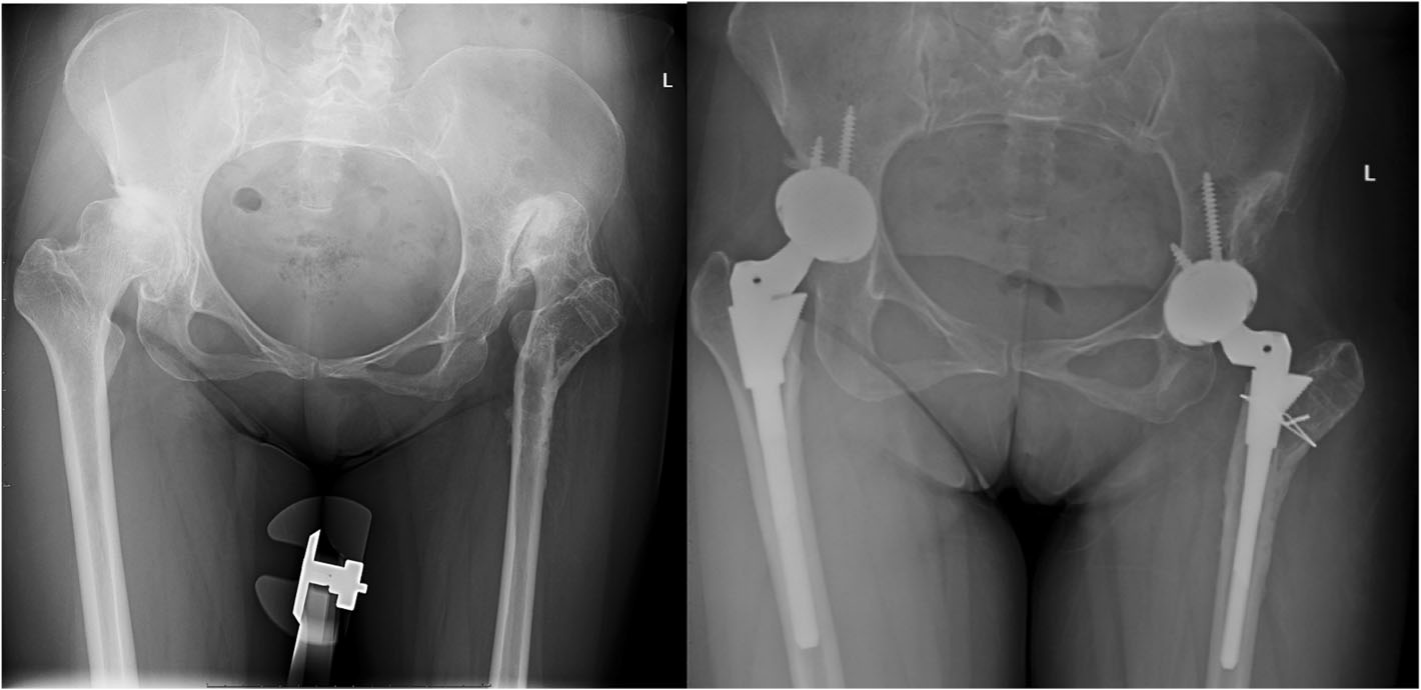
Female, 41 years. She underwent transtrochanteric valgus osteotomy 10 years ago

**Fig. 7:**
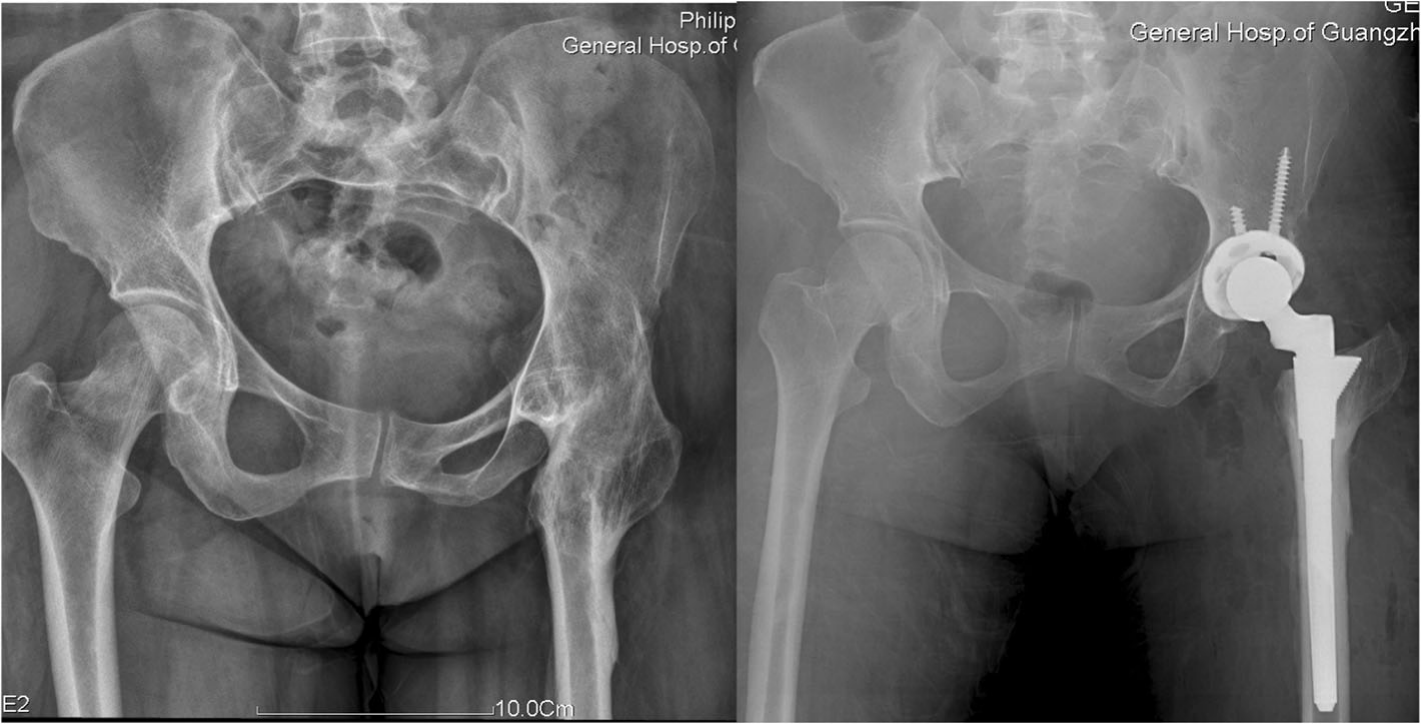
Female, 51 years. She underwent transtrochanteric valgus osteotomy 15 years ago

**Fig. 8:**
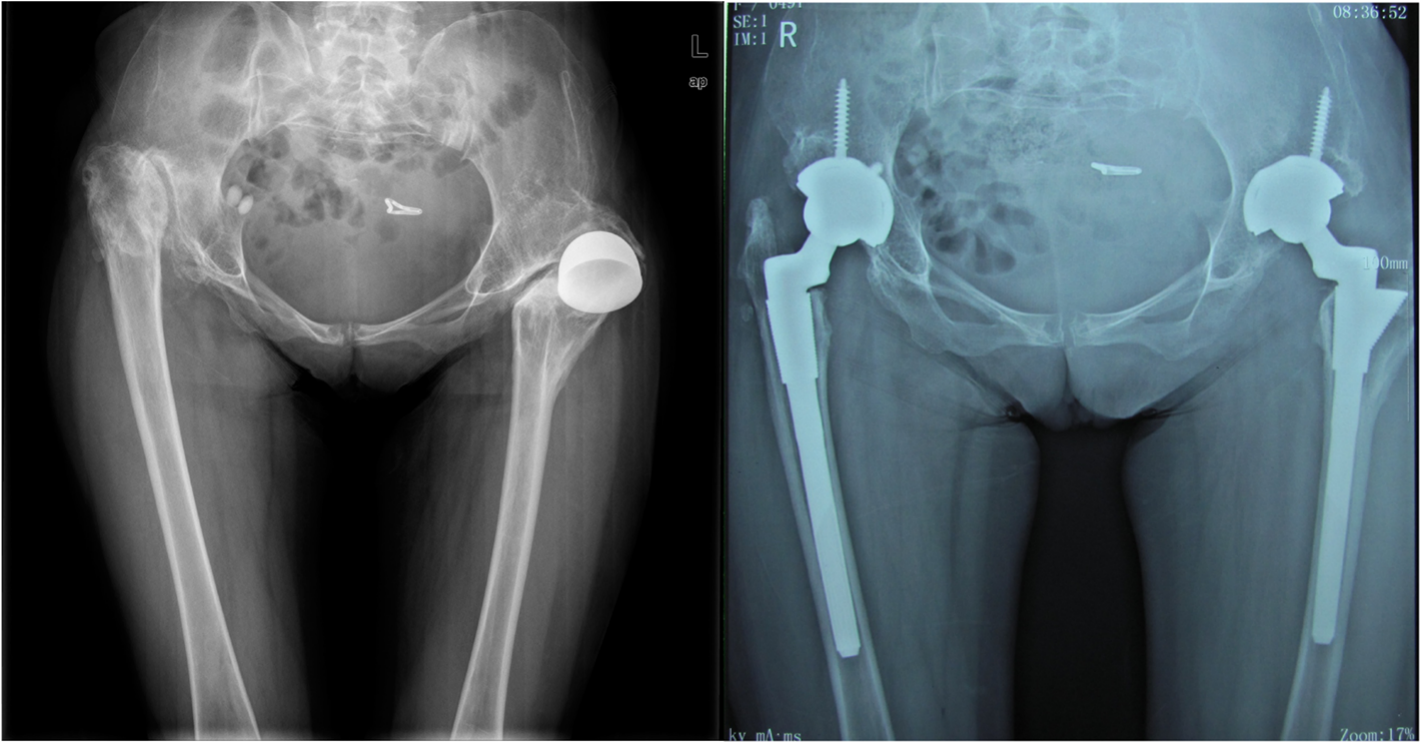
Female, 45 years. She underwent transtrochanteric valgus osteotomy 2 years ago

The typical cases were showed (Figs. 3, 4, 5, 6, 7, 8).

## Discussion

A numerous of hip-preserving surgeries were performed in young patients with hip dysplasia or ONFH [2, 13–15]. When these patients developed arthritis, the residual deformity would pose severe challenges to following THA [16]. The surgical strategy of THA varies along with the severity and position of femoral deformity [17]. As one uncommon type of femoral deformity, the severe valgus deformity of trochanter is critical for prosthetic morphology and surgical technique.

The concurrent arthroplasty with femoral osteotomy was a technically demanding procedure which had high risk of complications [1, 5]. Some surgeons suggested that customized prosthesis may provide one effective solution for severe femoral deformity [18–20]. However, the economic cost of customized prosthesis limited its wide application.

According to the design of S-ROM, the angle between sleeve and femoral stem can be adjusted freely, but it is seldom beyond 90 degree in clinical practice. The opposite direction of sleeve (180 degree) described in this study have never been reported previously.

In this study, no aseptic loosening or revision of femoral stem was found in the case series with a mean follow-up period of 6 years. There was the significant increase of HHS in all patients. The high revision rate of complex osteotomy or cemented THA was avoided. Better function and less complication indicated the safety and effectiveness of this technique in patients who had severe TVD. Cone has such advantages as easier bone preparation, less bone loss and less stress shielding. It was considered when an osteotomy is not planned, because the anti-rotation stability would be weakened by osteotomy [21, 22]. In the meanwhile, the risk of subsidence cannot be ignored in cone or fully-coated cylindrical stem [22, 23].

Although the method has produced satisfying clinical outcomes, we can’t neglect its technical flaws. Firstly, it can’t go through all kind of trochanter valgus deformities. Some special deformities still need osteotomy to facilitate offset and straighten medullary cavity. Secondly, since the valgus greater trochanter is not corrected, the increased joint offset would increase the risk of the greater trochanteric bursitis. Two patients reported lateral thigh pain after surgery, which was probably related to the bursitis. Thirdly, the manual work of implanting sleeve and malformed medullary cavity increased the risk of proximal femoral fractures. In this study, two patients had intra-operative fractures. Burs or other certain tools should be standing by for bone preparation. Fourthly, leg length might be influenced by residual deformity. While the equal leg length could be achieved by proper neck length and femoral head.

The clear indication of this special sleeve-implanting method was equal important. The medial support and lateral cover are two essential aspects of sleeve ingrowth. Once the medial cortex of proximal femur is destroyed, patients can’t meet the requirement of this special method. We can make basic predictions though measuring G/L ratio and TVA. In this study, compared with controls, the G/L ratio and TVA of 12 patients had significant differences, which indicated that their anatomies of proximal femur were characteristic. When G/L ratio was larger than 1.50, it can be regarded as one good indicator for the method.

Nowadays, an angular osteotomy on proximal femur is not suggested in hip-preserving surgeries. But this specific deformity was still occasionally met in conversion DDH for THA, which accounted for significant challenge. Although we introduced one alternative, it is necessary to remind the surgeons who are still performing angular osteotomy on proximal femurs of its potentially serious consequences.

This study has several limitations. Firstly, given that hip arthritis combining with trochanter valgus deformity were relative rare (12 cases in 9 years), suitable control cases could hardly be found to conduct case-control study. No comparison to other prostheses or other surgical methods would inevitably affect the persuasiveness of this study on technical notes. Secondly, because it was a retrospective case-series study, we don’t need the prospective ethics approval. In the future, multicenter randomized controlled trial will be performed to further evaluate its safety and effectiveness. Thirdly, this study was conducted over a long period of time. Changes in surgical personnel and related technical details might influence the final evaluation. Fourthly, study population be made up of various primary etiologies. The heterogeneity also had some impact on this method’s universality.

## Conclusions

The reverse sleeve of S-ROM was a reliable method for the patients with severe trochanter valgus deformity, which brought satisfying clinical outcomes in mid-term follow-up.

## Data Availability

The datasets used and/or analysed during the current study are available from the corresponding author on reasonable request.

## Abbreviations

DDH: Developmental dysplasia of hip
G/L ratio: Greater trochanter/lesser trochanter
GF_1_: Greater trochanter to the femoral axis
GF_2_: Lessor trochanter to the femoral axis
HA: Hydroxyapatite
HHS: Harris hip score
ICC: Interclass correlation coefficient
ONFH: Osteonecrosis of the femoral head
THA: Total hip arthroplasty
TVA: Trochanter valgus angle
TVD: Ttrochanter valgus deformity
TVO: Transtrochanteric valgus osteotomy
